# CpG Oligodeoxynucleotides Induce Differential Cytokine and Chemokine Gene Expression Profiles in Dapulian and Landrace Pigs

**DOI:** 10.3389/fmicb.2016.01992

**Published:** 2016-12-15

**Authors:** Jiaqing Hu, Dandan Yang, Hui Wang, Chuanhao Li, Yongqing Zeng, Wei Chen

**Affiliations:** Department of Animal Genetics and Breeding, College of Animal Science and Technology, Shandong Agricultural UniversityTai'an, China

**Keywords:** pig, CpG ODN, cytokines, chemokines, transcriptome

## Abstract

Oligodeoxynucleotides containing unmethylated CpG motifs (CpG ODN) mimic the immunostimulatory activity of microbial DNA by interacting with Toll-like receptor 9 (TLR9) to activate both the innate and adaptive immune responses in different species. However, few studies have been published to compare the effects of CpG ODN on different pig breeds. Therefore, in this study, whole blood gene expression profiles of DPL and Landrace pigs treated with CpG ODN were studied using RNA-seq technology. Five Hundred differentially expressed genes (DEGs) were identified between the two breeds. DPL pigs had significantly higher number of immune-relevant DEGs than the Landrace pigs after CpG ODN treatment. Pathway analysis showed that cytokine-cytokine receptor interaction and chemokine signaling pathway were the major enriched pathways of the immune-relevant DEGs. Further *in vitro* experiments showed that PBMCs of the DPL pigs had significantly higher levels of TLR9 mRNA than those of the Landrace pigs, both before and after CpG ODN stimulation. Cytokine and chemokine induction in the PBMCs of both breeds were also measured after CpG ODN stimulation. Our data showed that mRNA levels of cytokines (IFNα, IL8, IL12 p40) and chemokines (CXCL9, CXCL13) were significantly higher in the PBMCs of the DPL pigs than those of the Landrace pigs. Taken together, our data provide new information regarding the pig breed difference in response to CpG ODN stimulation and that higher levels of TLR9 mRNA in DPL pigs may be a major contributor for disease resistance.

## Introduction

In the pig production industry, infectious diseases caused by viral and bacterial pathogens seriously influence animal welfare, product quality and economics around the world (Meng, 2012). Breed is one of the most important factors that directly influence resistance or susceptibility to various infectious diseases (Reiner et al., 2002; Opriessnig et al., 2006; Lunney and Chen, 2010). Chinese indigenous pig breeds are generally better at disease resistance than foreign lean-type pig breeds (Yang, 2013). Dapulian (DPL) pigs are an indigenous breed of China, which mainly distribute in Shandong Province, China. Compared to modern commercial pig breeds, DPL pigs exhibit stronger resistance to diseases such as porcine reproductive and respiratory syndrome (PRRS) (Jiang et al., 2013; Xing et al., 2014). Since DPL and Landrace pigs were evolved under different environment and pathogen pressure, they may harbor variants of immune response genes which will help to explain their differences in disease resistance. Therefore, exploring the differential expression of immune response genes in DPL and Landrace pigs will help to understand their disease resistance difference on the molecular level.

Toll-like receptors (TLRs) are a family of pattern recognition receptors (PRR), which recognize invading pathogens by an array of conserved molecular structure known as pathogen-associated molecular patterns (PAMPs), playing critical roles in innate antimicrobial immune responses (Kumar et al., 2009; Takeuchi and Akira, 2010). TLR1, TLR2, TLR4, TLR5, and TLR6 are mainly present on the cell surface and recognize PAMPs derived from bacteria, fungi and protozoa; while TLR3, TLR7, and TLR9 are mainly present on the endosome and recognize PAMPs derived from various viruses and bacteria (Kumar et al., 2009; Takeuchi and Akira, 2010). Moreover, TLR agonists are known to induce intracellular signal transduction cascades and have been identified as therapeutic reagents for a number of diseases (McInturff et al., 2005; Uenishi and Shinkai, 2009). Among the TLRs, TLR9 has been shown to play important roles in host immune responses against a broad range of pathogens (Manuja et al., 2013). TLR polymorphism has been shown to be associated with disease resistance in human (Bhanothu et al., 2015), sheep (Swiderek et al., 2006), and chickens (Liu Y. et al., 2011). Recently it has been suggested that the higher mRNA levels of TLRs may explain why Tibetan pigs have stronger innate immunity compared to Yorkshire pigs (Cheng et al., 2015). Currently there is a lack of information about TLR signaling in DPL and Landrace pigs. Therefore, we hypothesized that differential TLR signaling in DPL and Landrace pigs may explain the difference in their innate and adaptive immune capacity.

Next-generation sequencing (NGS) technology has provided a very useful platform to easily compare DEGs and assess mRNA transcription patterns for all the genes in various species (Wilhelm and Landry, 2009). RNA-seq using NGS technology has become widely used for high-throughput researches of gene expression. Such technology has been successfully used to identify genes associated with economically important quantitative traits, such as body growth, immune, disease resistance, and meat quality (Mu et al., 2014; Sodhi et al., 2014; Ghosh et al., 2015; Xiao et al., 2015). In this study, we compared the gene expression difference in the whole blood samples of DPL and Landrace pigs 6 h after stimulation with class C CpG ODN 2429 (a TLR9 agonist) *in vivo* using RNA-seq technology. Based on the RNA-seq results, we further studied the mRNA expression of TLR9 and cytokine/chemokine in the peripheral blood mononuclear cells (PBMCs) of DPL and Landrace pigs after C CpG ODN 2429 stimulation *in vitro*. 500 DEGs were identified in the whole blood samples between the two pig breeds after CpG ODN stimulation and these DEGs' enriched pathways were also explored. Our data also suggest that higher level of TLR9 mRNA in the DPL pigs may contribute to stronger inflammatory reactions through higher cytokine/chemokine expression compared to Landrace pigs.

## Materials and methods

### Animals and ethics statement

Twelve female DPL and twelve female Landrace pigs (all at day 28 of age) were purchased from Jiaxiang DPL Farm, Jining City, China. These pigs were housed in adjacent pens (6 pigs per pen separated by breed) under the same standard conditions to reduce environmental effects on gene expression. The pigs had access to the same food three times a day and water *ad libitum*. All pigs were seronegative for antibodies to PRRS virus, porcine circovirus type 2 (PCV2), pesudorabies virus (PrV), classical swine fever virus and parvovirus as detected by commercial ELISA assays. All animal experiments in this study were conducted in accordance with the Institutional Animal Care and Use Committee of Shandong Agricultural University and the “Guidelines for Experimental Animals” of the Ministry of Science and Technology (Beijing, China).

### Gene expression analysis using RNA-seq

#### Animal treatment and blood sample collection

Six DPL and six Landrace pigs were injected with C CpG ODN 2429 TCGTCGTTTTCGGCGGCCGCCG (CpG) (Shenggong Biotech Co, Shanghai, China) at the dose of 500 μg/kg body weight according to the reference (Dar et al., 2010). The injection was performed on day 35 of age. Whole blood samples were collected from the external jugular vein at 6 h after injection. 1 mL whole blood samples were mixed with 3 ml RNALock Reagent (Tiangen Biotech Co, Beijing, China). The mixture was kept at room temperature for 2 h and then stored at −80°C until use.

#### cDNA library construction and RNA-seq

Among the 6 pigs from each breed treated with CpG ODN, blood samples from 3 DPL and 3 Landrace pigs were used for RNA-seq experiment. The whole blood RNA was extracted using a RNAsimple Total RNA kit (Tiangen Biotech Co, Beijing, China) according to the manufacturer's instructions. RNA concentration and purity were assessed using a NanoDrop 2000 Spectrophotometer (Thermo Fisher Scientific, Wilmington, DE, USA). RNA integrity was assessed using a RNA Nano 6000 Assay Kit on an Agilent Bioanalyzer 2100 system (Agilent Technologies, CA, USA). According to the manufacturer's recommendations, the sequencing libraries were generated using a NEBNext® Ultra RNA Library Prep Kit from Illumina (New England Biolabs, Ipswich, MA, USA) by Biomarker Technologies Corporation (Beijing, China). Briefly, mRNA was isolated from total RNA using magnetic oligo (dT) beads (Invitrogen, Carlsbad, CA, USA) and was further fragmented into about 200 bp. The first and the second strands of cDNA were synthesized using theses fragmented mRNAs. Finally, the suitable cDNA fragments were PCR-amplified to generate a complete cDNA library. The cDNA library was sequenced using an Illumina HiSeq 2500 instrument at the Biomarker Technologies Corporation (Beijing, China).

#### Analysis of RNA-seq data

After discarding low quality sequence reads (reads with adaptors, unknown nucleotides larger than 5%, or Q20 <20%) by perl script, all clean reads were aligned to the reference genome of *Sus scrofa* (*Sscrofa* 10.2; ftp://ftp.ensembl.org/pub/release-75/fasta/sus_scrofa/) using the TopHat2 software. The gene expression levels were calculated using the FPKM (fragments per kilobase of exon per million fragments mapped) values generated by the Cufflinks software (Trapnell et al., 2010). Genes (FPKM > 0.1) that were detected commonly in all six samples were assigned as co-genes. The DEGs were analyzed using the R package DESeq (Anders and Huber, 2010). The genes with Fold changes (log2Ratio) ≥2 and FDR significance score <0.01 were regarded as DE Gs. The false discovery rate (FDR) was used to evaluate the *p*-value in multiple tests. The *p*-value of the original hypothesis test was further corrected using the accepted Benjamini-Hochberg correction method.

All DEGs were submitted to the databases of Gene Ontology (GO) and Kyoto Encyclopedia of Genes and Genomes (KEGG) for enrichment analysis. GO analysis was performed using the Blast2 GO software (Conesa et al., 2005) to annotate the function of these DEGs. Moreover, the KEGG database was used for the DEGs enriched pathway analysis (http://www.genome.ad.jp/kegg/).

### TLR9 mRNA expression in the PBMCs of DPL and landrace pigs

#### Isolation of PBMCs and CpG ODN treatment

Whole blood samples from the remaining six DPL and six Landrace pigs also at day 35 after birth were collected via external jugular vein using 10 mL vacutainers coated with EDTA. PBMCs were isolated by FICOLL density gradient centrifugation as described by Fuss et al. (2009). Isolated PBMCs were re-suspended in RPMI 1640 medium supplemented with 10% feta bovine serum. PBMCs (1 × 10^6^ cells/mL) were stimulated with 5 μg/mL TLR9 ligand CpG ODN for 6, 12, and 24 h according to the reference (Auray et al., 2013). Control cells were cultured under the same condition except no TLR ligand treatment.

#### Gene expression assay using quantitative real-time PCR (qRT-PCR)

Total RNA of the PBMCs was extracted using Trizol (Takara Biotechnology, Dalian, China) according to the manufacturer's instructions. The quantity and quality of the isolated RNA were determined via UV260/280 using a biophotometer (Eppendorf, Hamburg, Germany). A two-step qRT-PCR Kit (Takara Biotechnology, Dalian, China) was used to generate cDNA according to the manufacturer's instructions. qRT-PCR was performed using a SYBR Premix Ex Taq kit (Takara Biotechnology, Dalian, China) on MX3000p RealTime PCR System (Stratagene, La Jolla, CA, USA). The primers (listed in Table 1) were either from published literature or designed in house using Premier 5.0 software (Applied Biosystems, Foster City, USA). B2M and PPIA genes were used as housekeeping genes (Wang et al., 2015). The gene expression differences were calculated using the 2^−ΔΔCT^ method (Livak and Schmittgen, 2001).

**Table 1:** **Primer sequences used in this study**.

**Primer name**	**Sequence (5′-3′)**	**Production size (bp)^a^**	**References^b^**
IFNα-F	CTGGCTGTGAGGAAATACTT	118	NM_001166318.1
IFNα-R	TTGTGGAGGAAGAGAAGACT		
IFNγ-F	GGAGCATGGATGTGATGAAG	137	Du et al., 2016
IFNγ-R	GAGTTCACTGATGGCTTTGC		
TLR9-F	GGCCTTCAGCTTCACCTTGG	151	Auray et al., 2010
TLR9-R	GGTCAGCGGCACAAACTGAG		
CXCL10-F	CTGTTCGCTGTACCTGCATC	232	NM_001008691.1
CXCL10-R	GCTTCTCTCTGTGTTCGAGG		
CXCL13-F	TTCTGGAGACCAATGACACA	172	XM_003129101.2
CXCL13-R	TGAGGGTTCAAGCAGATAGC		
CXCL9-F	TCAACACCAGCCAAAGGATG	180	NM_001114289.2
CXCL9-R	TGACCTGTTTCTCCCACTCT		
CCL19-F	TGCCAACGATGCTGAAGACT	231	NM_001170516.1
CCL19-R	TAGTTGCGGTGGTGCTTGCT		
MYD88-F	GGAACAGACCAACTATCGGC	126	Hu et al., 2016
MYD88-R	GAGACAACCACTACCATCCG		
CCL3L1-F	CTTCCTCGCAAATTCGTAGC	152	NM_001009579.1
CCL3L1-R	GCATTCAGCTCCAGGTCAG		
IL12 p40-F	GGGTGGGAACACAAGAGAT	154	Hu et al., 2016
IL12 p40-R	GGCTAAACTTGCCTAGAGGT		
B2M-F	TTCACACCGCTCCAGTAG	166	Martino et al., 2011
B2M-R	CCAGATACATAGCAGTTCAGG		
PPIA-F	CACAAACGGTTCCCAGTTT	171	Cinar et al., 2013
PPIA-R	TGTCCACAGTCAGCAATGGT		

*F, Forward primer. R, Reverse primer*.

a *Amplicon length in base pairs*.

b Genbank accession number of cDNA and corresponding gene, available at http://www.ncbi.nlm.nih.gov

### Statistical analysis

The expression of genes with ≥2-fold change and FDR adjusted *p*-value of <0.01 were considered significantly different in the RNA-seq results. qRT-PCR data were analyzed using one-way or two-way ANOVA with a Bonferroni post hoc test. *P* < 0.05 is considered significant.

## Results

### Overview of the sequencing data

In this study, we obtained approximately 31.4 gigabases (Gb) reads from the six RNA-seq libraries. After discarding low-quality reads, high percentage of the reads were mapped to the pig reference genome, ranging from 74.03 to 81.39%. Among the mapped reads, 69.74–78.96% were mapped uniquely to the pig reference genome. All six samples had nearly 90% reads equal to or exceeding Q30 (Table 2). Pairwise correlation was used to evaluate the individual variation in the blood samples. Except DPL1, all other five samples had very high Spearman correlations across all genes (ranging from 0.985 to 0.998). DPL1's Spearman correlation was very different from the other DPL sample (Spearman correlation for DPL1 across all genes only ranging from 0.474 to 0.460; Table 3). Therefore, DPL1 sample was excluded from further analysis The RNA-seq data were deposited into NCBI BioProject database, and its accession number is PRJNA309200.

**Table 2:** **A summary of the sequencing reads alignment to the *Sus scrofa* genome**.

**Sample**	**Landrace 1**	**Landrace 2**	**Landrace 3**	**DPL 1**	**DPL 2**	**DPL 3**
Total reads	48,632,150	39,206,506	42,966,768	32,416,978	44,590,578	43,659,992
Total base pairs	6,127,650,900	4,940,019,756	5,413,812,768	4,084,539,228	5,618,412,828	5,501,158,992
Mapped reads	39,582,267 81.39%	30,911,049 78.84%	33,909,206 78.92%	23,998,577 74.03%	35,345,365 79.27%	34,459,642 78.93%
Uniq mapped reads	38,400,961 78.96%	30,054,581 76.66%	32,696,054 76.10%	22,607,515 69.74%	33,916,617 76.06%	33,195,934 76.03%
%≥Q30	90.01	89.39	89.52	90.39	89.58	89.62
GC content %	58.36	58.84	58.07	55.94	57.91	58.14

**Table 3:** **Biology repeat correlation statistics**.

**Sample 1**	**Sample 2**	**r^2^**
L1	L2	0.9856
L1	L3	0.9946
L2	L3	0.9873
DPL1	DPL2	0.4738
DPL1	DPL3	0.4597
DPL2	DPL3	0.9985

### Identification and analysis of DEGs

**Figure 1:**
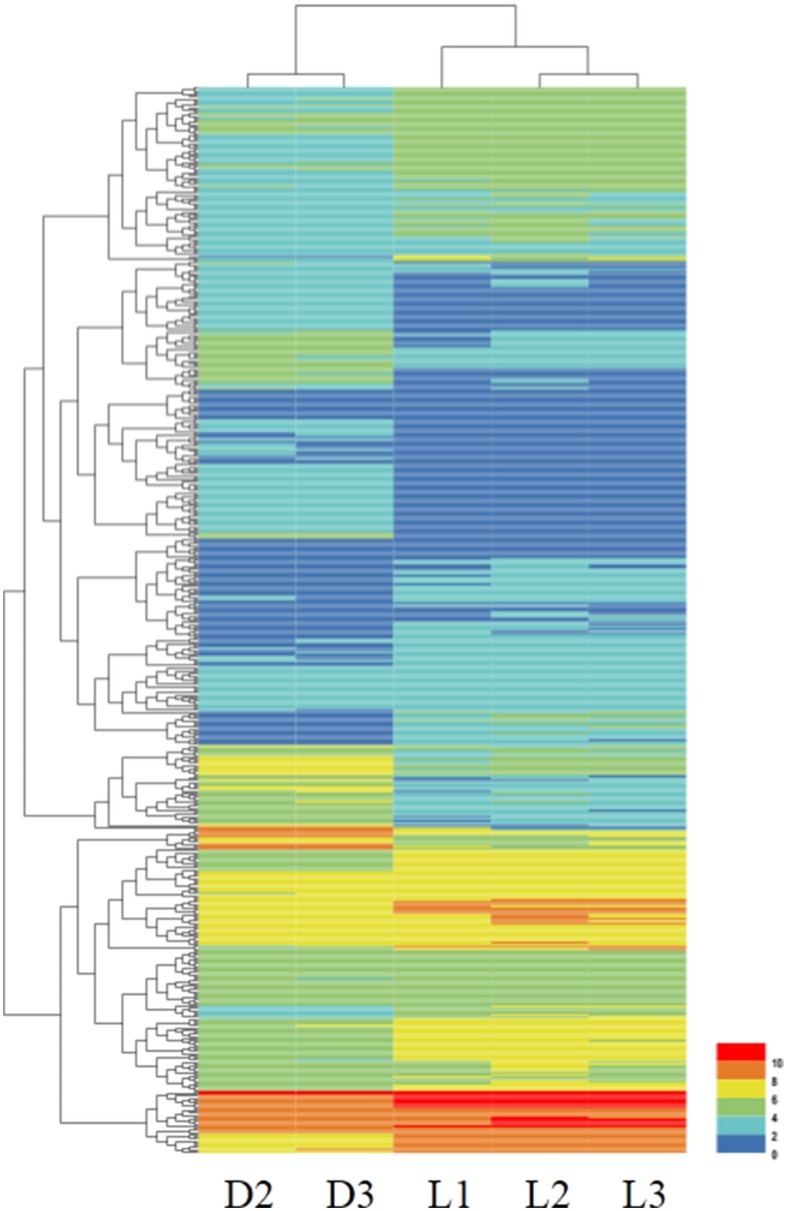
**Heatmap of the DEGs between the DPL and Landrace pigs**. Columns represent individual samples. Rows indicate genes with significant expression differences between the two breeds. The color represents the gene expression levels in the sample. From 0 to 10, the gene expression levels increase gradually.

After bioinformatics analysis, 500 genes were found to be differentially expressed in the whole blood samples of DPL and Landrace pigs. 64.2% (321 genes) of the DEGs had higher expression in the Landrace pigs, while 35.8% (179 genes) DEGs had higher expression in the DPL pigs. A heatmap of the DEGs was shown in Figure 1 and more detailed information of the DEGs was listed in Supplementary Table 1, including their log fold difference and FDR values.

### GO and KEGG pathway enrichment analysis of DEGs

**Figure 2:**
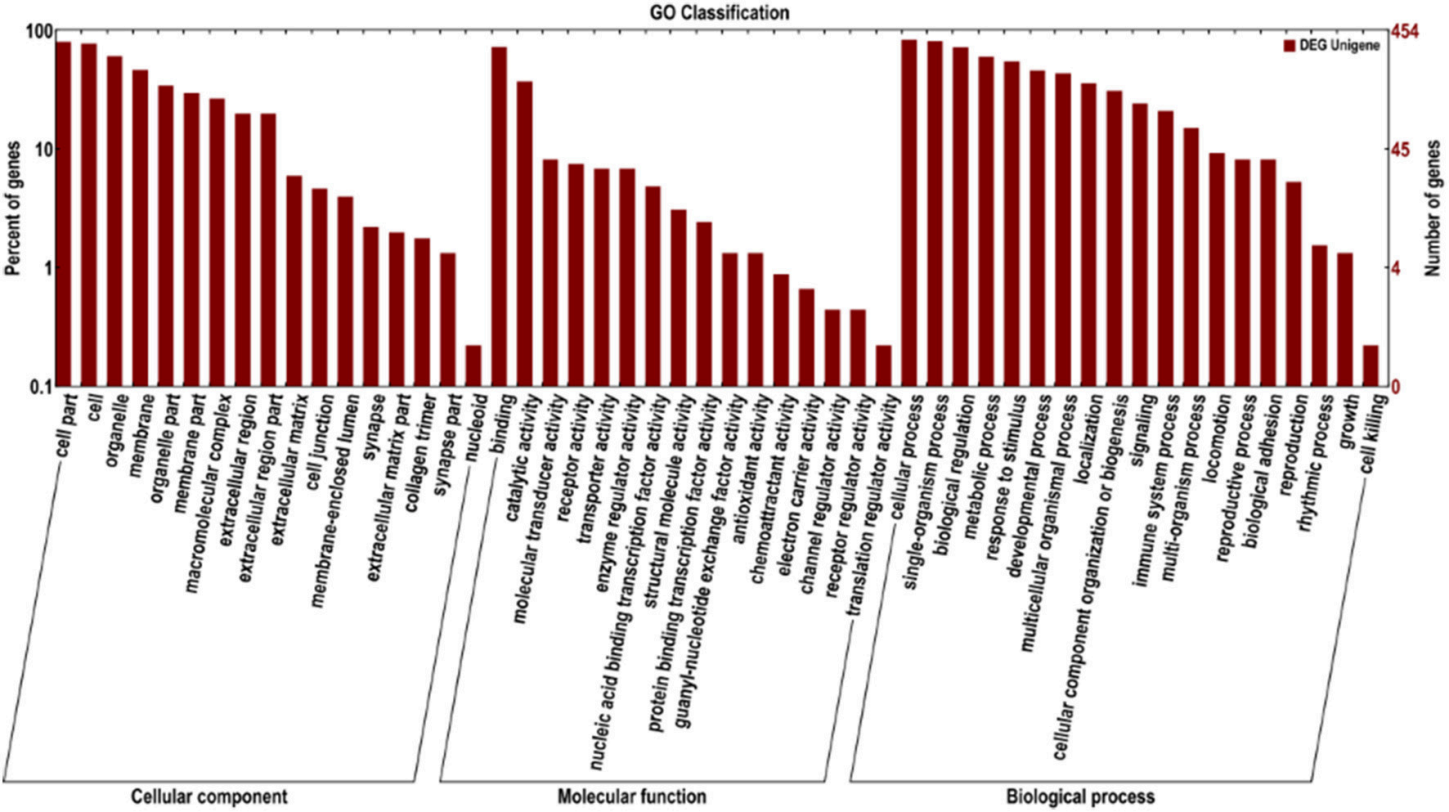
**GO analysis of DEGs between the DPL and Landrace pigs**. The DEGs are classified into three categories: cellular component, molecular function, and biological process. The percentage of genes in each category and the number of genes are shown above.

**Figure 3:**
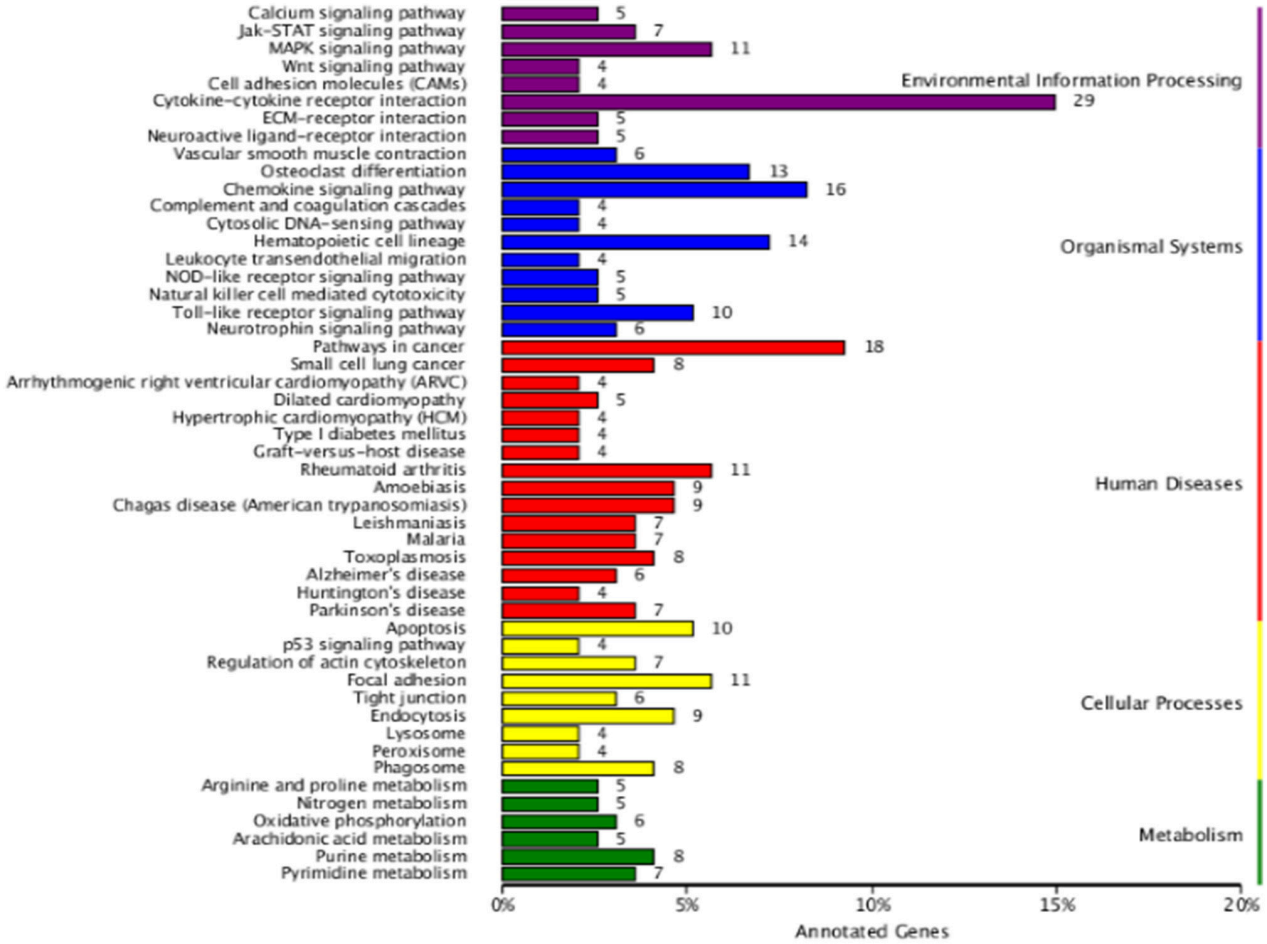
**KEGG analysis of DEGs between the DPL and Landrace pigs after CpG ODN stimulation**. The number of genes in each category are shown above.

To define the biological functions of the 500 DEGs, GO and KEGG analysis were performed. Fifty-two significantly enriched GO terms were identified. The main biological functions identified by GO analysis included molecular function, biological process and cellular component (Figure 2). The results have shown that the main functional groups of DEGs in cellular component were cell part, cell, organelle, and membrane; in molecular function were binding, catalytic activity; and in biological process were cellular process, single-organism process, biological regulation and metabolic process. Meanwhile, immune-relevant pathways were significantly enriched in the KEGG pathway analysis, including cytokine-cytokine receptor interaction, chemokine signaling pathway, MAPK signaling pathway, TLR signaling pathway, Jak-STAT signaling pathway, natural killer cell mediated cytotoxicity, and NOD-like receptor signaling pathway. The major pathways were listed in Table 4 and Figure 3.

**Table 4:** **The major enriched KEGG pathways analysis from DEGs in DPL and Landrace pigs**.

**Pathway**	**Ko ID**	**Genes**	
		**Down-regulated in Landrace**	**Up-regulated in Landrace**
Cytokine-cytokine receptor interaction	ko04060	TNFRSF18; IL12RB2; IL1B; IL1A; IL8; CXCL2; CXCL10; OSM; CCL19; CXCR6; CSF3; CCL4; CCL3L1; CCL2; CXCL9; CCR2; CCR5; CSF1; CXCL13; IL2RB	LTBR; CCL23; CCR2; KIT; CSF1R; TNFSF13
Pathways in cancer	ko05200	FOS; TRAF3; TRAF2; PLCG1; IL8; CASP3; NOS2; PIAP; JUN	MAX; BCL-XL; KIT; NCOA4; CSF1R; ITGA2B;
Chemokine signaling pathway	ko04062	IL8; CXCL2; CXCL10; CCL19; CXCR6; CCL4; CCL3L1; CCL2; CXCL9; CCR2; CCR5; CXCL13	FOXO3A; CCL23; CCR2
MAPK signaling pathway	ko04010	FOS; PLA2G2D; TRAF2; IL1B; IL1A; CASP3; DUSP4; JUN; Pig_newGene_8380	MAX; RPS6KA2
Toll-like receptor signaling pathway	ko04620	FOS; TRAF3; IL1B; IL8; CXCL10; CXCL9; JUN	ENSSSCG0000000900; MYD88; IRF5
Jak-STAT signaling pathway	ko04630	CCND2; IL12RB2; OSM; CSF3; ENSSSCG00000022485; ENSSSCG00000029668	BCL-XL

### DEGs associated with immunity

Immune relevant DEGs were significantly enriched in cytokine-cytokine receptor interaction and chemokine signaling pathway. It is worth noting that 82.8% of the immune relevant DEGs (24 out of 29) had higher expression in the DPL pigs compared to the Landrace pigs, such as IL1α, IL1β, CXCL8, CXCL10, CCL19, CXCR6 (The DPL/Landrace gene ratios were 3.33-, 1.74-, 5.63-, 2.24-, 5.55- and 3.55-fold respectively) and so on. Only 17.2% of the immune relevant DEGs (5 out of 29) had higher expression in the Landrace pigs compared to the DPL pigs, such as MyD88, CCL23, CCR2 and IRF5(the Landrace/DPL gene ratios were 6.1845-, 1.34-, 1.39-, and 1.20-fold respectively). The detailed information of the major immune relevant enriched genes were shown in Table 5.

**Table 5:** **Detailed information of the 29 major immune relevant DEGs in response to CpG ODN stimulation in DPL and Landrace pigs**.

**Sus scrofa Ensemble ID**	**Gene**	**FDR**	**Fold change (Landrace/DPL)**	**Regulation (Landrace/DPL)**	**Description**
ENSSSCG00000002525	TRAF3	0.004304	−1.26584	Down	TNF receptor-associated factor 3-like
ENSSSCG00000003330	TNFRSF18	1.02E-07	−2.01619	Down	Tumor necrosis factor receptor superfamily member 18-like
ENSSSCG00000003799	IL12RB2	0.000604	−2.13986	Down	Interleukin-12 receptor subunit beta-2 precursor
ENSSSCG00000005838	TRAF2	0.001752	−1.12747	Down	TNF receptor-associated factor 2
ENSSSCG00000008088	IL1B1	2.89E-06	−1.7407	Down	Interleukin-1 beta precursor
ENSSSCG00000008090	IL1A	0.003754	−3.32519	Down	Interleukin-1 alpha precursor
ENSSSCG00000008953	CXCL8	5.59E-11	−5.63162	Down	Interleukin-8 precursor
ENSSSCG00000008959	CXCL2	0.007887	#NAME?	Down	C-X-C motif chemokine 2 precursor
ENSSSCG00000008977	CXCL10	2.09E-12	−2.24248	Down	C-X-C motif chemokine 10 precursor
ENSSSCG00000009469	IRG1	8.15E-05	−2.5073	Down	Immune-responsive gene 1 protein homolog
ENSSSCG00000010966	CCL19	1.48E-11	−5.54945	Down	C-C motif chemokine 19 precursor
ENSSSCG00000011318	CXCR6	1.98E-05	−3.54542	Down	C-X-C chemokine receptor type 6
ENSSSCG00000014441	CSF1R	0.007496	1.315574	Up	Macrophage colony-stimulating factor 1 receptor
ENSSSCG00000016573	IRF5	0.001758	1.201847	Up	Interferon regulatory factor 5-like isoform 1
ENSSSCG00000017698	CCL4	1.79E-06	−2.35038	Down	C-C motif chemokine 4 precursor
ENSSSCG00000017700	CCL3L1	1.17E-12	−2.73058	Down	C-C motif chemokine 3-like 1 precursor
ENSSSCG00000017702	CCL23	0.0012	1.341277	Up	C-C motif chemokine 23-like
ENSSSCG00000017723	CCL2	0.00608	−4.09919	Down	C-C motif chemokine 2 precursor
ENSSSCG00000021847	SAA4	3.61E-71	−6.49546	Down	Serum amyloid A-4 protein-like
ENSSSCG00000022737	Myd88	0.006187	6.18454	Up	Myeloid differentiation primary response protein MyD88-like
ENSSSCG00000023489	CXCL9	0.005054	#NAME?	Down	C-X-C motif chemokine 9 precursor
ENSSSCG00000024270	CCR2	0.001995	#NAME?	Down	C-C chemokine receptor type 2
ENSSSCG00000024311	CCR2	0.000314	1.396261	Up	C-C chemokine receptor type 2
ENSSSCG00000024344	CCR5	2.58E-05	−1.62968	Down	C-C chemokine receptor type 5
ENSSSCG00000024914	CFB	1.68E-06	−4.21557	Down	Complement factor B precursor
ENSSSCG00000026832	CSF1	0.000255	−2.19443	Down	Macrophage colony-stimulating factor 1 precursor
ENSSSCG00000028731	CXCL13	5.59E-11	−6.87266	Down	C-X-C motif chemokine 13-like
ENSSSCG00000028875	HAVCR2	1.58E-15	−3.98527	Down	Hepatitis A virus cellular receptor 2-like
ENSSSCG00000029668	IL2RB	5.34E-08	−1.75442	Down	Interleukin-2 receptor subunit beta-like

### qRT-PCR validation of the gene expression data from RNA-seq

**Figure 4:**
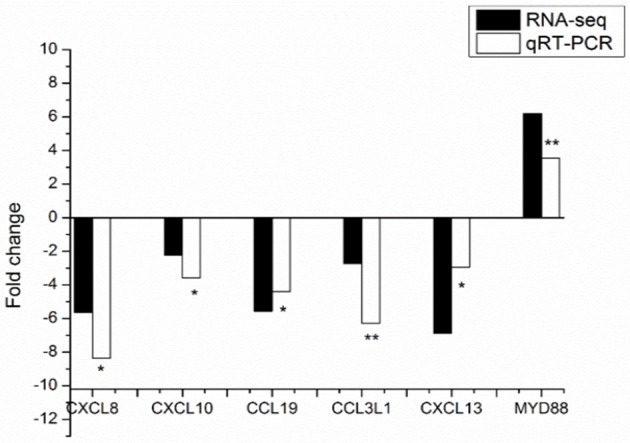
**Validation of 6 identified DEGs from RNA-seq results using qRT-PCR**. Positive and negative values are either up or down regulated genes in comparisons (Landrace/DPL). qRT-PCR data were calculated by the 2^−ΔΔCT^ method. The direction and magnitude of the fold changes obtained using the qRT-PCR technique were similar to those of the RNA-seq data (^*^p < 0.05, ^**^p < 0.01). *p*: Student's *t*-test *p*-values between DPL and Landrace. ^*^Significant (*p* < 0.05) difference, ^**^extremely Significant (*p* < 0.01) difference.

To verify the gene expression data by the RNA-seq, qRT-PCR was performed for six biologically important genesfor immunity, including one gene with lower expression level and five genes with higher expression levels in the Landrace pigs compared to the DPL pigs. qRT-PCR validation was performed using blood samples from all the pigs injected with CpG ODN except DPL1 (i.e., 5 DPL and 6 Landrace pigs injected with CpG ODN). Gene expression fold changes of Landrace/DPL in qRT-PCR analysis and RNA-seq were shown in Figure 4 and Table 6. The expression patterns of the six genes were generally consistent with the RNA-seq results, suggesting that the results of the RNA-seq experiments were accurate and reliable.

**Table 6:** **The mean ± SD of the DPL and Landrace pigs validated by qRT-PCR. *p*: Student's *t*-test *p*-values between DPL and Landrace**.

**Gene**	**Myd88**	**CXCL8**	**CXCL10**	**CCL19**	**CCL3L1**	**CXCL13**
DPL	0.93 ± 0.07	1.32 ± 0.36	1.19 ± 0.33	1.13 ± 0.34	1.14 ± 0.23	1.23 ± 0.22
Landrace	3.09 ± 0.59	0.16 ± 0.02	0.33 ± 0.13	0.26 ± 0.11	0.18 ± 0.05	0.42 ± 0.14
*p*-value	*p* = 0.0095	*p* = 0.0116	*p* = 0.0405	*p* = 0.0387	*p* = 0.0047	*p* = 0.0224

### TLR9 mRNA expression in the DPL and landrace PBMCs

**Figure 5:**
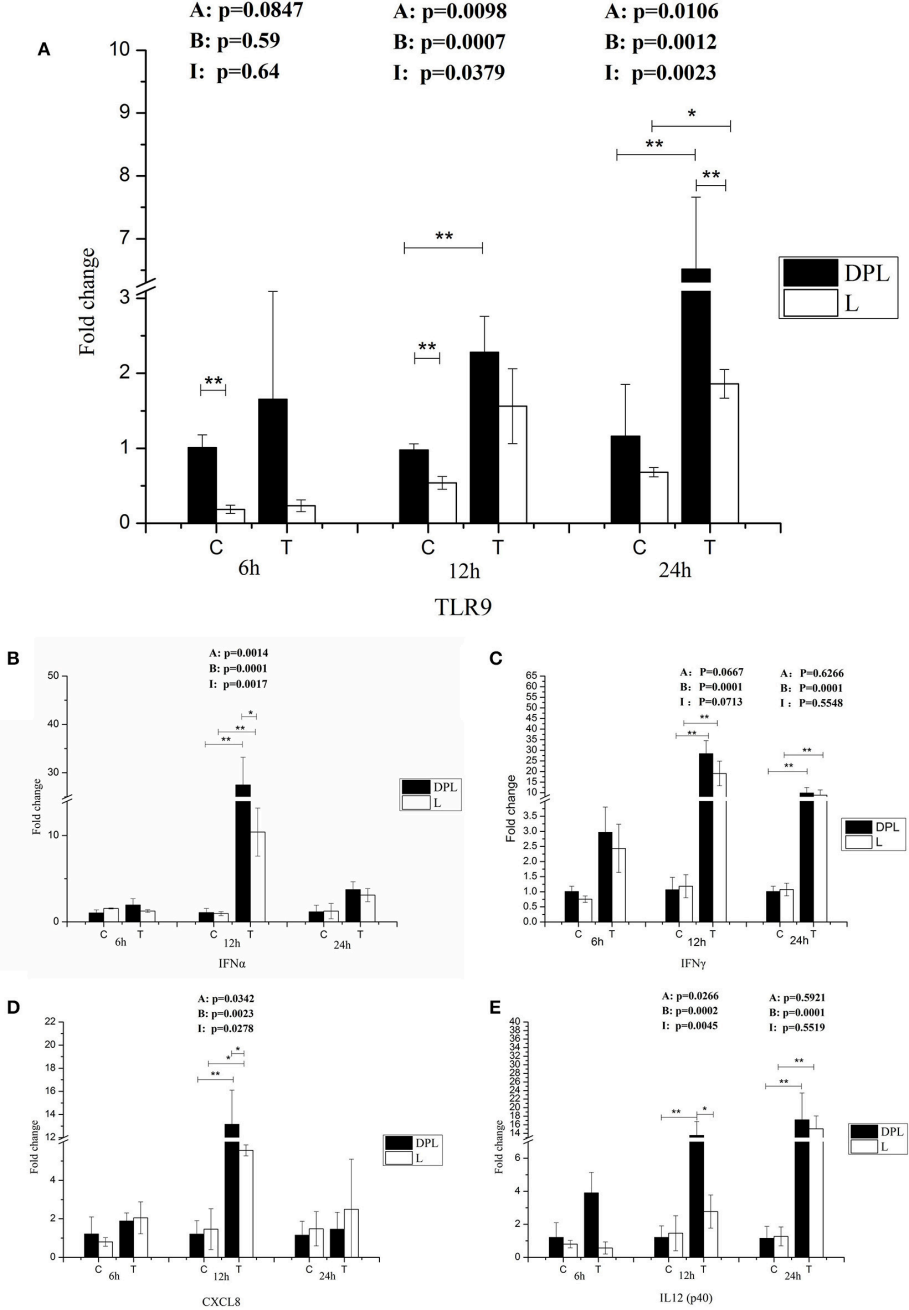
**TLR9 and cytokines gene expression in DPL and Landrace PBMCs after CpG ODN treatment**. Gene expression of TLR9 and cytokines in stimulated DPL and Landrace PBMCs was assessed using qRT-PCR, the data were calculated by the 2^−ΔΔCT^ method with B2M and PPIA as housekeeping genes. **(A)** TLR9, **(B)** IFN_α_, **(C)** IFN_γ_, **(D)** CXCL8, **(E)** IL12 p(40). At the each time point of 6, 12, and 24 h, the un-stimulated DPL and Landrace pig PBMCs samples as negative control. Data represent the mean ± SD from 6 different animals (^*^p < 0.05, ^**^p < 0.01, *Post-hoc* test: Bonferroni-Holm). A, (Factor A = Breed); B, (Factor B = treatment); I, (Factor I = breed × treatment). C, control; T, treatment; L, Landrace; DPL, Dapulian.

Because CpG ODN is an agonist for TLR9, we tested TLR9 gene expression in DPL and Landrace PBMCs with or without treatment with CpG ODN at different time points (Figure 5A). Without CpG ODN treatment, the DPL pigs had significantly higher mRNA levels of TLR9 than the Landrace pigs at 6 and 12 h (DPL/Landrace ratio 5.44- and 1.81-fold respectively); TLR9 mRNA levels in the DPL PBMCs treated with CpG ODN increased significantly at 12 and 24 h compared to control DPL PBMCs (2.33-, 5.61-fold); while TLR9 mRNA levels in the Landrace PBMCs increased significantly only at 24 h compared to the control Landrace PBMCs (2.7-fold). Furthermore, with CpG ODN treatment, the TLR9 mRNA level was significantly increased in the DPL PBMCs compared to the Landrace PBMCs at 24 h (3.51-fold). The data suggest that the DPL pigs had higher TLR9 mRNA levels in the PBMCs compared to Landrace pigs at early time points without CpG ODN treatment. TLR9 mRNA expression was more responsive to CpG ODN treatment in the DPL PBMCs compared to the Landrace PBMCs (based on the fact that the mRNA expression of TLR9 in the DPL PBMCs increased earlier than the Landrace PBMCs, and the expression level of TLR9 mRNA in the DPL PBMCs was significantly higher than that of the Landrace PBMCs).

### Cytokine gene expression in the DPL and landrace PBMCs

Because large number of cytokine/chemokine genes were differentially expressed based on the RNA-seq result, we further studied their gene expression in the PBMCs of DPL and Landrace pigs after CpG ODN stimulation. The expression levels of IFNα and IFNγ genes were significantly higher after 12 h stimulation with CpG ODN in both breeds (25.47-, 10.89-fold); moreover, the gene expression levels of IFNα was significantly higher in the DPL than the Landrace pigs at 12 h (2.64-fold) (Figures 5B,C). IL8 (CXCL8) mRNA level was significantly increased in both DPL and Landrace breeds after stimulation with CpG ODN at 12 h (10.89-, 3.80-fold). The DPL pigs had significantly higher IL8 mRNA level than the Landrace pigs at 12 h after stimulation with CpG ODN (2.37-fold). Two-way ANOVA demonstrated a significant effect of both breed and CpG ODN treatment on IL8 gene expression, and there was also a significant interaction between these two factors on IL8 gene expression (Figure 5D). After stimulation at 12 h, the expression level of IL12 p40 gene was significantly higher in the DPL pigs than the Landrace pigs (4.89-fold) (Figure 5E). Two-way ANOVA demonstrated a significant effect of both breed and CpG ODN treatment on IL12 p40 gene expression; and there was a significant interaction between these two factors on the expression of IL12 p40 gene. The data suggest that both breed and CpG ODN treatment had significant effect on TLR9 signaling.

### Chemokine gene expression in the DPL and landrace PBMCs

**Figure 6:**
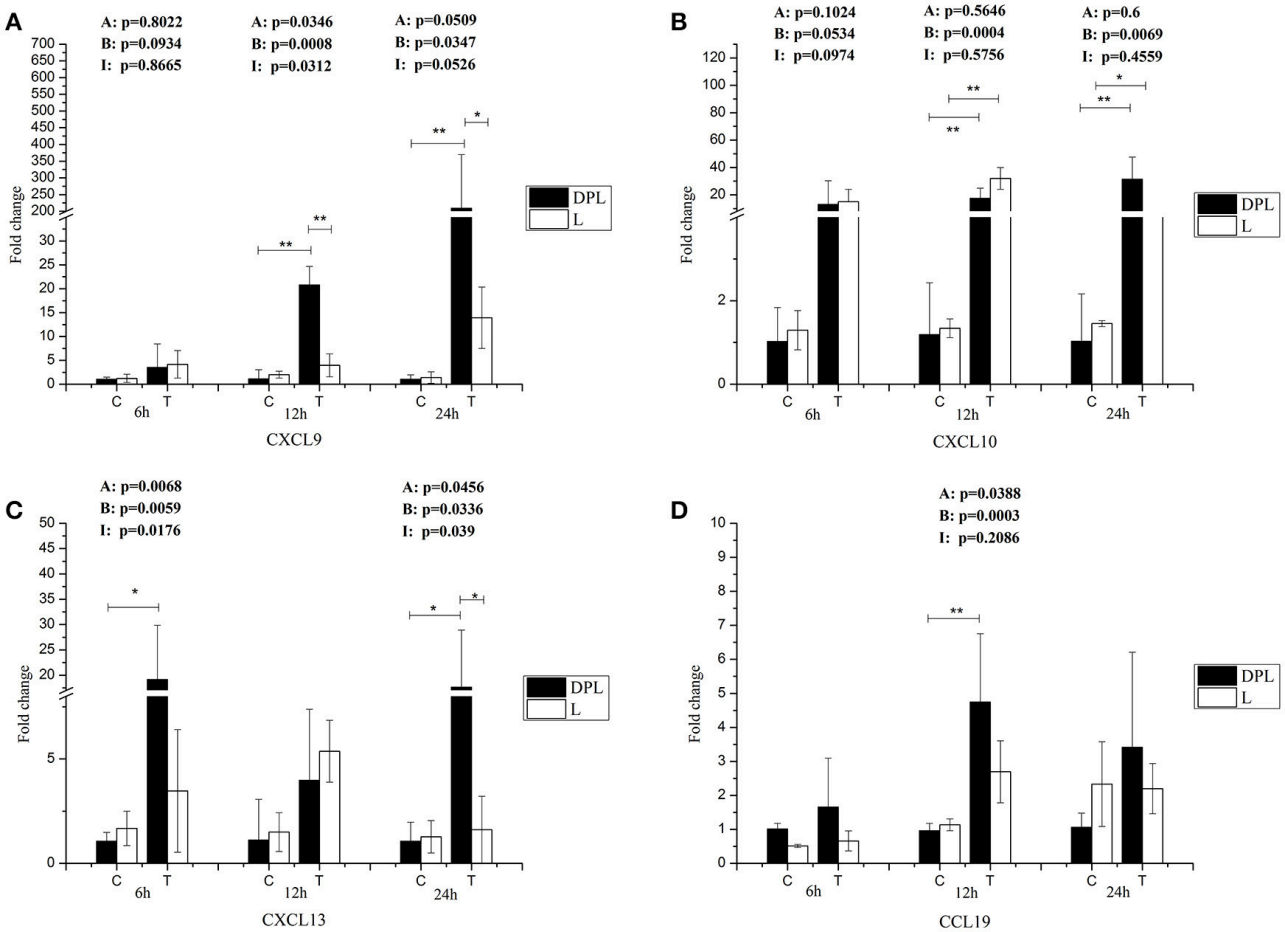
**Chemokine genes expression in DPL and Landrace PBMCs after CpG ODN treatment**. Gene expression of chemokine in stimulated DPL and Landrace PBMCs was assessed using qRT-PCR, the data were calculated by the 2^−ΔΔCT^ method with B2M and PPIA as housekeeping genes. **(A)** CXCL9, **(B)** CXCl10, **(C)** CXCL13, **(D)** CCL19. At the each time point of 6, 12, and 24 h, the un-stimulated DPL and Landrace pig PBMCs samples as negative control. Data represent the mean ± SD from 6 different animals (^*^p < 0.05, ^**^p < 0.01, *Post-hoc* test: Bonferroni-Holm). A, (Factor A = Breed); B, (Factor B = treatment); I, (Factor I = breed × treatment). C = Control; T = treatment; L = Landrace; DPL = Dapulian.

After stimulation with CpG ODN, the expression levels of CXCL9 and CXCL13 genes were significantly higher in the DPL pigs compared to the Landrace pigs at 24 h (15.00-, 10.9-fold; Figures 6A,C). After stimulation with CpG ODN, both breeds had significantly increased levels of CXCL10 gene at 12 h (39.77-, 43.38-fold) and 24 h (29.75-, 13.06-fold) for DPL and Landrace respectively (Figure 6B). CCL19 gene was significantly higher in the DPL pigs 12 h after stimulation (4.93-fold). However, no significant CCL19 gene expression change was found in the Landrace pigs at any time points with or without CpG treatment (Figure 6D).

## Discussion

In this study, the transcriptional differences in the whole blood samples of DPL and Landrace pigs after a TLR9 agonist treatment were studied both *in vivo* and *in vitro*. CpG ODN has been used previously to stimulate pig immune system (Auray et al., 2010; Dar et al., 2010). Overall, there were significant differences in the whole blood gene expression between DPL and Landrace pigs. For example, the RNA-seq results showed that there were 500 DEGs in the whole blood between these two breeds after CpG ODN stimulation. The large number of DEGs identified suggest that the DPL and Landrace pigs responded to CpG ODN stimulation quite differently. Many identified DEGs were immune-relevant, which was in agreement with the immunostimulatory effect of CpG ODN treatment. Further bioinformatics analysis showed that these DEGs were enriched in the cytokine and chemokine signaling pathways, suggesting that cytokines and chemokines were important mediators for TLR signaling. Most immune-relevant DEGs had higher expression in the DPL pigs compared to the Landrace pigs (82.8 vs. 17.2%). The large proportion of immune-relevant DEGs with higher expression in the DPL pigs suggest that DPL pigs elicit a stronger immune reaction after CpG ODN treatment, which may help to explain its stronger disease resistance.

We next studied TLR9 gene expression in the PBMCs of DPL and Landrace pigs treated with CpG ODN *in vitro*. TLRs are critical components of the innate immune system. Moreover engagement of these receptors may affect the type of acquired immunity. It has been shown that TLR polymorphism or their expression level may confer disease resistance in different animal species or different pig breeds (Uenishi et al., 2011; Yang et al., 2013). The activation of TLRs by different agonists have been shown as potential therapeutic reagents for a number of diseases (McInturff et al., 2005). Toll-like receptors are the best characterized pattern recognition receptor and are recognized as the major sensors of pathogens. After activation by TLR agonist, the nuclear factor-κB (NF-κB) was activated to regulate immune response and inflammation through MyD88 dependent or independent pathways (Barton and Medzhitov, 2003; Kawai and Akira, 2010). Therefore, understanding the molecular mechanism of TLR activation pattern in different pig breeds will provide a great promise for porcine disease resistance genetic improvement. TLR9 recognizes unmethylated CpG motifs, and then induces the recruitment of MyD88 to initiate downstream signaling (Takeshita et al., 2004). Furthermore, it has been shown that TLR9 associates with MyD88 to initiate CpG ODN mediated effects via signal-transducing pathways including NF-κB, p38 MAPK, JNK, AP-1, and ATF-2 (Akira et al., 2001; Osawa et al., 2006; Zhang et al., 2013). PBMCs express a wide range of TLRs and are crucial targets for various pathogens. The TLR9 ligand CpG ODN was shown to provide protection against infectious diseases (Manuja et al., 2013). Our data showed that TLR9 mRNA had a higher expression level in the DPL pigs compared to the Landrace pigs without CpG ODN treatment *in vitro*. TLR9 mRNA levels in the DPL PBMCs were more responsive to CpG ODN treatment than the Landrace PBMCs. Even though the Landrace TLR9 mRNA was significantly increased at 24 h post CpG ODN treatment, it was still significantly lower than the DPL pigs. These results suggest that the two breeds have differences in sensitivity to CpG ODN treatment. The higher level of TLR9 mRNA and its responsiveness to CpG ODN treatment in DPL (a pig breed resistant to PRRS) is in agreement with literature showing that higher TLR levels were associated with disease resistance (Oliveira et al., 2004; Karnati et al., 2014; Cheng et al., 2015). Surprisingly, TLR9 mRNA level was not found to be significantly different between the two breeds in the RNA-seq dataset, which was not in agreement with the PBMCs *in vitro* results. A possible explanation is that the *in vitro* experimental condition for PBMCs was different from the *in vivo* whole blood because PBMCs missing many medium present in the plasma. Moreover, the intercellular relationship between immunocompetent cells may have changed after PBMC separation. For example, it has been shown that the gene expression levels were different in the whole blood and PBMCs after stimulation with agonists (Groote et al., 1992).

MyD88 is an essential adapter for TLR9 signaling (Takeshita et al., 2004). Our data showed that the Landrace pigs had significantly higher expression of Myd88 mRNA in the whole blood than the DPL pigs. Previous studies have shown that there were great variability in gene mRNA expression in the same tissues of different pig breeds (Yu et al., 2010; Sodhi et al., 2014). Substantial inter-individual variation between pigs and the small sample number in this study may also influence the DEGs identified. Moreover, in our study, we only compared the RNA-seq data with the CpG ODN stimulation, lacking the data without CpG ODN treatment in the two breeds. Therefore, there is a possibility that DPL pigs have higher baseline TLR9 signaling.

Cytokines are cell-signaling proteins, which play crucial roles in haematopoiesis, inflammation and clearance of pathogens (Turner et al., 2014). IFNα, a type I interferon, is a key cytokine of the host innate immune system to control virus infection (Biron, 1998). IL8, also known as CXCL8, which plays important roles in the recruitment of neutrophils, chemotactic migration and activation of monocytes, lymphocytes, basophils, and eosinophils at the sites of inflammation (Turner et al., 2014). In addition, IL12 p40 is a subunit of IL12 but also of IL23, plays a critical role in cell-mediated immunity (Oppmann et al., 2000; Vignali and Kuchroo, 2012). In this study, we showed that the gene expression levels of IFN α (2.64-fold), IL8 (2.37-fold), and IL12 p40 (4.89-fold) were significantly higher in DPL than Landrace pigs after stimulating PBMCs with CpG ODN. Other studies showed that the gene expression levels of IFN α, IL8, and IL12 p40 were significantly higher after stimulation with CpG ODN or infection with pathogens in swine (Dar et al., 2010; Borghetti et al., 2012; Liang et al., 2016). Therefore, the significantly higher levels of theses cytokines in DPL pigs may play crucial roles in DPL disease resistance.

Chemokines are a group of small (8–12 kDa) proteins, and characterized by cysteine residues and separated into two groups depending on the presence (C-X-C family) or absence (CC family) of an intervening amino acid between cysteine residues (Turner et al., 2014). In the chemokines we studied in the PBMCs, CXCL9 (15.00-fold), and CXCL13 (10.9-fold) genes had significantly higher levels in the DPL pigs than the Landrace pigs, which is in agreement with the higher TLR9 levels in the DPL pigs. CXCL9 is a T-cell chemoattractant (Gorbachev et al., 2007; Hertenstein et al., 2011), which is secreted by all kinds of cell types including neutrophils, monocytes, endothelial cells and fibroblasts (Taub et al., 1993). Studies have shown that in the setting of disease or stimulation with pre-inflammatory cytokines, the level of CXCL9 was increased (Coma et al., 2006; Ohta et al., 2008). C-X-C motif chemokine 13 (CXCL13), also known as B lymphocyte chemoattractant is a homeostatic chemokine that is constitutively expressed in secondary lymphoid tissue and involved in lymphoid organogenesis (Carlsen et al., 2004). When in pathological status of adult-onset Still's disease, the production of CXCL13 was up-regulated, and it was regarded as a crucial chemokine in the establishment of the adaptive immune response in the pathogenesis (Han et al., 2015). Therefore, the significantly higher levels of CXCL9 and CXCL13 mRNA in the DPL pigs after stimulation with CpG ODN may play important roles in DPL disease resistance. CXCL10 was significantly increased in the two breeds after stimulation, which is agreement with other studies showing that the CXCL10 is a reliable biomarker for immune activity induced by CpG ODN in pigs (Dar et al., 2010). Moreover, Landrace pigs seem to be more responsive at 6 and 12 h, while DPL pigs seem to be more responsive at 24 h. Due to the individual variation, no significant differences was found in the two breeds for CXCL10 gene *in vitro* after stimulation. The significantly higher levels of CXCL10 in the DPL pigs may play an important role in DPL disease resistance. Previous studies have shown that CXCL10 is a reliable biomarker for immune system activity induced by CpG ODN in pigs (Dar et al., 2010). Moreover, alterations in CXCL10 expression levels have been associated with inflammatory diseases including infection, immune dysfunction and tumor development (Liu M. et al., 2011). Therefore, CXCL10 is also recognized as a biomarker that predicts severity of various infections (Liu M. et al., 2011). Our results imply that TLR9 may elicit a stronger immune reaction through the induction of the above cytokines/chemokines. Hence, further studies are needed to explore the downstream and upstream signaling pathways regulating chemokines and their receptors ligands, particularly CXCL10 and their receptor ligands CXCR3, with the aim to exploit new methods to control infectious diseases mediated by theses chemokines.

To summarize, our data suggest that DPL pigs exhibited more responsiveness to CpG ODN stimulation than Landrace pigs. Regulating TLR9 mRNA levels in pigs might be a novel way to improve their immune capacity. The TLR9 mRNA levels may be regulated either using pharmacological methods or by guided breeding to select pig breeds with higher TLR9.

## Author contributions

YZ, WC, and JH designed this study. JH, DY, and WC took samples and performed the experiments, and analyses. YZ, WC, JH, and HW write and revised this manuscript. JH, DY, and CL modificated the manuscript.

### Conflict of interest statement

The authors declare that the research was conducted in the absence of any commercial or financial relationships that could be construed as a potential conflict of interest.
